# China and EU’s wisdom in choosing competition soft law or hard law in the digital era: a perfect match?

**DOI:** 10.1007/s12689-023-00101-8

**Published:** 2023-04-13

**Authors:** Kena Zheng, Francis Snyder

**Affiliations:** 1 Maastricht University, Maastricht, The Netherlands; 2 Peking University School of Transnational Law, Peking University Shenzhen Graduate School, Shenzhen, China; 3 CERIC, Aix-Marseille University, Aix-en-Provence, France; 4 University of Macau, Macau, China; 5 College of Europe, Brugge, Belgium

**Keywords:** Competition law, Soft law, Hard law, Digital markets

## Abstract

The development of digital technologies has led to the emergence of new business models benefiting consumers in their searching, shopping and communicating activities. However, it also challenges the applicable competition law framework and enforcement. Although there seems to be a global consensus on the need to update competition law, the EU and China have chosen different regulatory tools—hard law and soft law, respectively—to address the new and complex challenges facing the competition governance of digital markets. This paper aims to explore why the EU opted for hard law while China opted for soft law, and to further examine whether the selected regulatory tool is the most appropriate in the specific context. By analyzing and comparing characteristics of the digital market and competition institutions’ powers in the EU and China, the paper concludes firstly, that, through the use of hard law, the EU is able protect the Internal Market and at the same time overcome the inflexibility of hard law by adding review articles. Secondly, the paper concludes that, through the use of soft law, China is able to take advantage of soft law to rapidly respond to public attention in the digital market and, at the same time, overcome the non-legally binding force of soft law by strengthening the power of its competition authority.

## Introduction

Over the past decades, the information and communication technology (ICT) sector has undergone rapid development, from microelectronics in the 1940s, to the birth of the computer in the 60 s, and the introduction of the internet in the 90 s.^1^ A new economy enabled by digital technologies has emerged,^2^ such as E-Commerce, Fintech, Artificial Intelligence (AI) and robotics. Specifically, digitalization has opened vast prospects for business innovation, which helps to create new models and opportunities for business as well as more innovation for products and services. Moreover, the COVID-19 pandemic has accelerated digitalization processes. More and more people have continued, to the extent possible, with their activities through online channels for working, studying, communication, selling and buying, or entertainment.^3^ Despite the many benefits that digital innovation has brought, however, much of the enthusiasm and idealism that were so characteristic of the early years of the Internet has given way to concerns and skepticism.^4^

One of the principal concerns of economic regulation in the era of digital economy is to facilitate fair competition in markets.^5^ Part of this concern is a fear that the digital sector will naturally favor the growth of hugely influential platforms and ecosystems that will dominate the economy and use their power to extend their influence on social and political issues.^6^

Competition law is aimed at maintaining the competitive dynamics also in the digital economy and has brought concrete and important changes for market players and consumers.^7^ Nevertheless, although competition doctrine and theory traditionally have prided themselves on being attuned to economic realities, they have lagged behind these rapid changes in the economic landscape.^8^ Only within the last few years have economists and legal scholars started working to develop a methodology for identifying the competitive injuries that platform-based environments enable.^9^ It is possible to summarize those challenges for competition law into two aspects—the legal framework and law enforcement.

There is a gap between the current competition legal framework and the development of digital markets. A growing body of literature suggests that many concepts and tools derived from traditional business models do not generally apply to digital companies, as online platforms have complex business models and arrangements.^10^ The existing regulatory toolkit may not be enough to scrutinize data-driven algorithmic models.^11^ For instance, secondary heuristics such as the competition regulator’s basic distinction between horizontal and vertical integration strategies do not translate well to the platform-based environment. Thus, the adoption of new regulations and tools can complement the current competition legal framework.^12^ On the other hand, the development of the digital economy also brings challenges to competition law enforcement. Officials from the United States, UK, France and Germany have publicly recognized the potential harm and question the adequacy of their current enforcement tools. The complexity and opacity of platform-based, massively intermediated exchange structures have stymied courts and policymakers used to working with more traditional economic models.^13^ The ability to intervene quickly is recognized as an absolute necessity in digital markets.^14^

Generally, there are two basic regulatory tools: hard law and soft law. Most modern legal regimes adopt a conscious hybrid system in competition law with a mix of hard and soft instruments, to pursue the same objective.^15^ Several states and regions have realized the significance of updating competition law in the digital era. But finding a balanced and optimized governance approach is not going to be easy, as the issues at hand are complex.^16^ Some competition agencies now routinely issue guidelines and staff interpretations to signal regulated entities about their interpretations of governing status and rules and their likely enforcement stances.^17^ However, several states and regions have chosen to apply hard law in Big Tech competition.^18^

The EU and China have chosen different tools for regulating competition in digital markets. The EU published and proposed two hard law instruments—*Regulation on Promoting Fair and Transparency for Business Users of Online Intermediation Services* (*P2B Regulation*) on 11 July 2019 to promote fairness and transparency for business users of online intermediation services, and the *Digital Market Act* (*DMA*) on 1 November 2022 to promote contestable and fair markets in the digital sector by targeting “gatekeepers” which have become an important gateway for business users to reach end users due to strong positive network effects and economies of scale.^19^ China published instead a soft law instrument named Anti-*Monopoly Guidelines for the Platform Economy* (*Platform Guidelines*) in February 2021 to strengthen antitrust enforcement in the Internet platform sector and to provide guidance for tech companies.^20^

In light of these developments, the purpose of this paper is to answer the two questions: first, why the EU and China have chosen different regulatory tools – hard or soft law, to regulate competition in the digital market; secondly whether the selected regulatory tool is the most appropriate in the specific context. It is structured in five parts. Part 2 compares, in general, soft law and hard law to understand their advantages and disadvantages. Part 3 discusses why the EU chose hard law and China chose soft law to regulate the digital market. Part 4 compares two jurisdictions to understand their choice of regulatory tools further. Then the last part gives some conclusions.

## Differences between hard and soft law

Soft law instruments are part of a reality that is difficult to ignore nowadays, as hybrid modes of regulation that combine soft and hard law instruments are put in place in many sectors,^21^ including competition.^22^ For instance, Stefan testifies that the EU competition domain is defined by the hybridity of (legal and non-legal) instruments the Union issues, whereby ‘soft law adds further precision to the general rules provided for in the Treaty, thus specifying and concretizing the law’.^23^ Nevertheless, soft law comes in an “infinite variety”,^24^ which presents a challenge for any attempt at defining, classifying, or developing a framework of analysis.^25^ Soft law is an oft-used concept, which is still given different meanings as no consensus has emerged in scholarship.^26^ The most quoted academic definition of soft law is by Snyder, who defines soft law as “rules of conduct which, in principle, have no legally binding force but which nevertheless may have practical effects and also legal effects”.^27^

This section continues by summarizing three advantages, two debated issues and a disadvantage of soft law as regulatory tool.

### Three advantages of soft law

Soft law might be preferable to hard law in some circumstances. Trubek et al. classified seven general explanations for why soft law might be preferable to hard law.^28^ Based on their conclusions, we summarize three rather uncontroversial advantages of soft law, which are to some extent overlapping.

1. Lower negotiation costs. A major advantage of soft forms of governance is their lower negotiation or “contracting” costs, especially when the issues are unfamiliar or complex.^29^ Compared to hard law, soft law can reduce the levels of obligation, delegation and precision, making cooperative agreements possible.
2. Flexibility. The extensive use of soft law can be seen as a reaction to the need for a more flexible regulatory system.^30^ Firstly, the greater flexibility of soft law allows for renegotiation or modification as circumstances evolve over time.^31^ Soft law is well equipped to cope with new problems by providing the flexibility necessary to allow for the possibility of required reform that can accommodate diverse legal systems.^32^ Secondly, soft law can take divergent circumstances into account through flexible implementation, which in turn helps to deal with the domestic political and economic consequences.^33^ Lastly, soft law also is appropriate when it is impossible to specify a precise standard.^34^ It offers more effective ways to deal with uncertainty, especially when it initiates processes that allow actors to learn about the impact of agreements over time.^35^ In comparison, hard law approaches are relatively inflexible.^36^ Hard law cannot be revised or supplemented frequently due to the need for legal certainty.^37^
3. Simplicity and speed. Soft law obeys a logic of ‘flexible governance’,^38^ with the simplicity of formulation and modification procedures. It does not need to be drafted in such a careful manner as hard law,^39^ enabling it to meet a need for faster regulation. Due to the less formal adoption process, it can also be less expensive than traditional legislation. In addition, it has been underlined that the simplified adoption procedures for soft law are highly valued characteristics in regulating sensitive sectors in which agreements are very hard to reach, tackling issues of regulatory philosophy and broad policy, and addressing situations where swift action is imperative.^40^ Soft law is also useful when there is a need to reach agreements quickly.^41^ For example, several soft law instruments were adopted on a contingency basis during the COVID period.^42^ However, the establishment of hard law must strictly comply with statutory procedures often including fundamental rights provisions whose structures are generally rigid.^43^ The procedures of hard law are formal and complex, which makes it slow to cope with uncertainty and contingency.

In sum, the advantages of soft law satisfy the regulatory requirements in the rapid development of information technology and the digital economy. It is preferred to provide guidance and solutions for complex issues, and solve problems with the diversity and mutability of social relationships and affairs stimulated by digitalization and technology.^44^

### Two debates on using soft law

Despite the advantages discussed above, the use of soft law still sparks debates.

1. More participation in law-making? In principle, soft law permits the integration of all interested parties in the process of law-making. Increased openness allows for more active participation of non-state actors, promotes transparency, enhances agenda setting, and facilitates the diffusion of knowledge.^45^ Some scholars suggest that the use of soft law can promote social power,^46^ as both the formulation and implementation of soft law depend largely on proactive communication and negotiation.^47^
  However, soft law making may bypass the legislature’s involvement and normal accountability systems.^48^ It is more natural for the executive arm of government who was increasingly used to power for over a century, to seek to avoid dealing with an obstructive parliament and cumbersome procedures.^49^ Thus, there is also a concern for the possible expansion of administrative power, which may lead to the abuse of powers.^50^ If, for instance, the European Commission lacks the power to enter into a legally binding commitment, it could instead use soft law to achieve the same result.^51^ The European Parliament has criticized that the Commission issued soft law to avoid the legislative procedure through the Parliament and the Council.^52^
  No conclusions have been drawn about whether the soft law-making procedure increased more interested parties is better or more important than the legislative procedure led by legislatures.
2. Promotion of legal certainty? Legal certainty is always believed as one of the intrinsic advantages of hard law. It is, on the whole, wise legal policy to use hard rules as much as possible for regulating human behavior because they are more certain and accessible to the public, at least in theory, and lend themselves more easily to uniform and predictable application.^53^
  Legal scholarship generally assumes that tightly specified rules increase legal certainty.^54^ However, when a more complex and changing phenomenon is being regulated, soft rules can regulate a transitional technology with more certainty than fixed hard rules.^55^ For example, Andrew Simpson makes the case that the regulation of international technologies like telecommunications requires flexible rules. What a telephone means today may be something quite different tomorrow.^56^ Soft law, likes a stop-gap measure, provides an initial foundation for future legislation to increase legal certainty.^57^ Soft law then may represent a first step on the path to legally binding agreements,^58^ which functions as a springboard in the development of hard law.^59^ Meanwhile, soft law can also be seen as a halfway solution, a non-binding instrument with a guiding role,^60^ to help the public to understand hard rules.^61^ Thus, soft law instruments are considered to protect legal certainty on complicated issues, especially buttressed by obligatory principles.^62^

### Disadvantages of soft law

While soft law has drawn increasing attention, it has not received uniform support.^63^ In general, soft law has vague legal consequences. It, in principle, has no legally binding force and is not enforced by state coercive power.^64^ That was considered as ‘fatal’ drawback because the enforcement of soft law might be difficult. Despite the fact that soft law may have legal and practical effects and encourage consistency as well as disseminate information,^65^ it mainly relies on social coercion or self-discipline mechanisms.^66^ For instance, Trubek et al. outline that shaming is one of the ways for EU soft law to affect and change behavior. Member States seek to comply with guidelines to avoid negative criticism in peer review and the Council recommendations.^67^

However, hard law takes advantage of its legally binding force in enforcement with coercive power, forcing actors to consider the threat of sanctions.^68^ The legal consequences of hard law are certain and clear, which makes it easier for private actors to predict the effects of their behaviors.

## The EU and China’s choices of different instruments

According to the United Nations Conference on Trade and Development, the goal of policymakers and legislators is to maximize the potential positive impacts of the digital economy, ensuring that these benefits are widely shared and minimizing the negative effects.^69^ This section analyzes the EU’s choice to adopt hard law and China’s choice to adopt soft law from two perspectives. One is to explore the benefits and negative effects for the European and Chinese digital markets. Furthermore, the powers of competition institutions, in particular the function of competition enforcement authorities, are discussed to explain the effectiveness of hard and soft law in two jurisdictions.

### The EU’s choice of hard law

The 1957 *Treaty of Rome* first established the European Economic Community’s competition regime, which has evolved alongside the unique supranational entity, the EU.^70^ Competition law lies at the heart of the EU legal framework for creating and supervising of the Internal Market. Before the challenges of the digital market have come to the fore, the EU competition provisions are mainly contained in Articles 101 and 102 of the *Treaty on the Functioning of the European Union* (*TFEU*) and in the *EU Merger Regulation*.^71^ Competition law is playing an ever-increasing role within the EU, due to the incredibly large fines imposed in the context of the digital market.^72^

#### The European digital market

The digital transformation is one of two large-scale challenges for Europe, along with the green transformation.^73^ In the new strategy *Shaping Europe’s Digital Future* launched in February 2020, the Commission voiced its commitment to taking an active role in the digital transformation. By 2030, the cumulative additional GDP contribution of new digital technologies could amount to 2.2. trillion euros in the EU, a 14.1 per cent increase from 2017.^74^ The Union has already realized that digital services and products have brought important innovative benefits to the Internal Market.

Three facts can be underlined to distinguish the European digital market from others. Firstly, the European digital market has a cross-border nature. Digital players typically operate across several Member States. Almost 24 per cent of total online trade in Europe is cross-border.^75^

Secondly, it is, however, American tech firms that hold the majority of the European market share. Although EU has realized that capturing the full value of the digital transformation requires implementing digital innovation and establishing a strong international position through world-leading digital research and companies, Europe has only attracted 13 per cent of global venture capital and corporate funding to increase spending for digital technologies and AI start-ups.^76^ More importantly, Europe is currently dependent on foreign-developed and -owned technology assets for a significant part of its digital economy.^77^ For instance, a large number of horizontal search engines offer search services in Germany.^78^ However, Google answered the vast majority of search queries (91 per cent). Similar spreads were reached in other European countries such as France.^79^ Equally, according to Statcounter, Google has taken 92.96 per cent of the search engine market share; Facebook has taken 80.22 per cent of the social media market share in 2021.^80^ At the same time, the Commission explained that over 10,000 online platforms operate in Europe’s digital economy, mostly start-ups and smaller businesses (SMEs).^81^ SMEs are also negatively impacted by this situation, “as it impedes them from scaling-up and from cross-border expansion, thereby reaching new markets, offering better and diversified products at more competitive prices and, as the case may be, growing into challengers of established players in the digital sector”.^82^ Thus, DG Competition aims to directly regulate Big Tech companies, also known as gatekeepers, although the Regulatory Scrutiny Board^83^ and several scholars argue that the initiative does not fully justify the gatekeepers and selection of the core platform services by DG Competition.^84^

Lastly, legal uncertainty is faced by the digital market players.^85^ Digital players typically operate at a global scale and deploy global business models. Several Member States have already started introducing new rules to address concerns arising from the presence of challenges.^86^ Even those Member States who have not yet adopted legislation are increasingly considering national measures to that effect.^87^ Fragmentation is also observable due to differences in the legislations in the internal market. For instance, the circle of potential addresses of the *DMA* appears to be wider than the Section 19a of *German Competition Act*,^88^ in particular in view of the qualitative criteria, which may trigger a presumption that the firm may be designated as a gatekeeper.^89^ As a result, different legislations within the EU may lead to increased regulatory fragmentation and increased compliance costs for these large market players and the business users that rely on them.^90^ Regulatory initiatives by Member States, however, cannot fully address these effects; without action at the EU level, they could lead to a fragmentation of the Internal Market.^91^

All these facts attracted the EU’s attention. On the one hand, the EU has published several digital strategies to promote innovation of European businesses. As all sectors are on the point of being radically reshaped by the combination of connectivity and data, and digital technology is at the heart of this transformation, Europe may want to reduce its dependency on foreign technology.^92^ On the other hand, the Union, under the principle of subsidiarity embodied in Article 5(3)-(4) *Treaty of European Union (TEU)*, believes that competition in the digital market cannot be sufficiently achieved by the Member States by reason of the scale or effects of the proposed action, and can therefore be better achieved at the Union level.^93^ Notably, the EU plans to issue new rules to protect fair competition in the Internal Market. The Commission’s focus on digitization in general and algorithms, in particular, is evident from a number of speeches by Mrs Vestager.^94^ However, what can the Commission do to protect fair competition in the digital economy? The next part will analyze powers of EU competition institutions.

#### The power of competition institutions

The EU is not a state, but rather a regional integration scheme of sovereign states, and is hence a divided-power system, based on the principle of attributed competences and powers.^95^ Two types of power balance need to be analyzed in competition law. On the one hand, the powers at the EU level among the Commission, the Parliament, the Council and the Court of Justice of the European Union (ECJ). On the other hand, the powers between the EU and the Member States, such as the Commission and the national competition authorities (NCAs).

##### Powers at the EU level

The power of the European institutions is formally governed by Treaty provisions.^96^ The separation of powers can be understood from legislators, competition authorities and judicature aspects in the fields of competition law.

1. Legislators. According to Article 14(1) *TEU*, the Parliament shall, jointly with the Council, exercise legislative power in the EU. The Ordinary Legislative Procedure in Article 294 *TFEU* asks the Commission to submit the proposal to the Parliament and the Council. Then the Parliament and the Council shall adopt their positions at three readings and communicate them to the Commission. The emphasis throughout the procedure is on compromise and dialogue to facilitate the successful passage of the legislative act.^97^ Furthermore, in the area of competition law, Article 103 *TFEU* provides that the Council must, after receiving a proposal from the Commission and consulting the Parliament, lay down the appropriate legislation to give effect to the principles set out in Articles 101 and 102 *TFEU*.^98^ The Council has adopted several pieces of legislation that are paramount in EU competition law.^99^
2. Competition authority. The Commission plays a leading role in developing and enforcing EU competition law.^100^ Above all, the Commission has the power to prosecute cases and to adopt decisions requiring the termination of infringements of the rules on competition and imposing fines.^101^ Article 17(1) *TEU* stipulates that the Commission shall ensure the application of the Treaties and measures adopted by the institutions pursuant to them. It shall exercise coordinating, executive and management functions laid down in the Treaties. More importantly, Article 105 *TFEU* highlights that the Commission shall ensure the application of the principle laid down in Articles 101 and 102. In addition to its power to deal with individual cases, the Commission also acts to develop a given practice on its own initiative by issuing soft law,^102^ such as notices and guidelines. These soft approaches indeed have self-binding effects to limit the Commission’s discretion.^103^
3. Judicature. Article 19(1) *TEU* states that the ECJ shall ensure that the law is observed in the interpretation and application of the Treaties. Although several pieces of research were conducted to show soft law has been taken into consideration in concrete cases,^104^ there is no obligation for the ECJ to apply soft law. Furthermore, Article 263 *TFEU* stresses that the ECJ shall review the legality of legislative acts, of acts of the Council, of the Commission, other than recommendations and opinions, and acts of the Parliament and of the Council intended to produce legal effect vis-à-vis third parties. Under the Article, the ECJ has jurisdiction to supervise the legal acts and considers that competition institutions in question must not exceed its power.^105^ But there is no requirement for reviewing soft law instruments and indeed the case law has been very restrictive when it comes to judicially reviewing EU soft law measures.^106^

##### Powers between the EU and member states

When *Regulation 17* was adopted in 1962,^107^ it was decided that the Commission should become the central competition authority in the Community (now Union). Since 1 May 2004, the system of centralized notification and prior authorization by the Commission under Article 101 *TFEU* was abolished.^108^*Regulation 1/2003* delegated competition enforcement power to NCAs and national courts in order to relieve the Commission of its increasing administrative burden and make enforcement more effective.^109^ More importantly, *Regulation 1/2003* increased the necessity for adopting more soft law instruments.^110^ Commission-initiated soft law supposedly diminishes the threat to consistent enforcement resulting from multi-level setting. This makes soft law indispensable for national enforcers, especially where formal Commission decisions do not sufficiently inform national decisional practice.^111^

The viability of the enforcement regime proposed in the Regulation on Procedure depends on close cooperation and coordination between NCAs and the Commission.^112^ Because NCAs are bound by Commission decisions which should incorporate the more economic reasoning of the guidelines, they are most likely also going to adopt a more economic reasoning.^113^

Besides, national courts have ‘an essential part to play’ in applying the competition rules.^114^ The role of the national courts has become more prominent in recent years as a result of their ability to award damages for breaches of Articles 101 and 102 *TFEU*, both for breaches established by a prior finding of a competition authority (including the Commission) and for breaches first established by the national court.^115^ However, the national courts’ attitudes to soft law are different. Some would like to take soft law into account,^116^ while some take a vague attitude.^117^ In fact, national courts could stray away from the guidelines because more economic soft law is not necessarily aligned with the case law of the supranational courts, which jurisprudence national courts are obliged to follow.^118^ If this scenario comes to fruition, NCA decisions would not be upheld on appeal.^119^

#### Analysis

Hard law would be better for competition institutions to act in accordance with the power conferred by the Treaties, to secure the institutional balance and guarantee democracy within the EU.^120^ In contrast, soft law issued by the Commission may avoid of the participation of the Parliament and the Council, and not be applied indirectly by the ECJ. On the other hand, Member States may also prefer to apply hard law. The powers of the Union are conferred by the Treaties agreed with all Member States. As the Treaties clarifies that soft instruments are of non-legally binding force, the NCAs and national courts would not be responsible for enforcing them like hard law. The legal practices showing different attitudes to soft law enforcement by NCAs and national courts in different Member States, could also prove that the enforcement of hard law is more effective than soft law in the EU.

Thus, if the goal is to promote an internal digital market in the EU, harmonization (of the regulation aimed at tech firms) via hard law seems the most appropriate choice for the EU. Indeed, the EU recently published the *P2B Regulation* and the *DMA*. In its *DMA Proposal*, the Commission stressed that:

Only a legislative instrument can effective address the problems identified. A Regulation is in addition necessary, as it is directly applicable in Member States, establishes the same level of rights and obligations for private parties, and enables the coherent and effective application of rules in the inherently cross-border online intermediated trade generated in the online platform economy.^121^

Additionally, in order to promote flexibility of hard law to respond to the rapid development of digitalization and technology, the EU added the review articles. For instance, Articles 4 (2) and 53 asks the Commission to review and evaluate the effectiveness and efficiency of measures every three years, to ensure that the *DMA* remains up to date and constitutes an effective and holistic regulatory response to the problems posed by gatekeepers.^122^

### China’s choice of soft law

The Chinese law-makers believe that competition law is a crucial component of a legal system required to support the functioning of a market economy.^123^ They understood that competition law would help to establish competitive market structures, promote and ensure efficiency of economic development.^124^ Indeed, while competition law intervenes only when there is a market failure, the competition agencies sometimes leveraged the law as a tool to fulfil its mission to maintain market stability.^125^

#### The Chinese digital market

China possesses one of the world’s largest and most vibrant digital economies.^126^ While overall digitalization is still lagging behind advanced economies, China has emerged as a global leader in key industries.^127^ Furthermore, China has experienced rapid digitalization in recent years. The size of the digital economy has surged from 15 per cent of GDP in 2008 to 33 per cent in 2017, mainly driven by the integration of ICT with traditional sectors.^128^

In fact, the Chinese government has already realized the significant role of digitalization and technology in developing the economy. According to the *14th Five-Year National Information Plan* published by the Chinese Cyberspace Administration on 27 December 2021,^129^ China’s digital economy ranks second in the world. Leading digital platforms from China—namely Alibaba, Baidu and Tencent—also benefited, experiencing altogether a net income increase of 37 per cent, from almost $20 billion in 2017 to $27 billion in 2019. The increase in profits was even more remarkable in 2020, as the cumulative net income was approximately $48 billion, a rise of 78 per cent compared with 2019.^130^ Last but not least, the Chinese digital market is dominated by native tech firms. For instance, Baidu took over 78 per cent of the market share in the search engine market.^131^

However, some cases have emerged in China in a few years, such as ‘choose one from two’ between Alibaba and Pinduoduo,^132^ and algorithmic price discrimination cases of several travelling platforms.^133^ These cases have triggered massive public concerns and posed a threat to social stability. Since 2020, the Chinese government’s attitudes to the platform economy have changed from ‘tolerant and cautious’ to ‘harshness’, which greatly influences China’s regulatory process in competition law.^134^ Several significant central-level conferences have proposed strengthening competition regulations and resolutely opposing monopolistic behaviors. The Politburo meeting held on 11 December 2020 called for strengthening anti-monopoly and preventing the disorderly expansion of capital.^135^ The Central Economic Work Conference on 16 December 2020 listed strengthening anti-monopoly as one of the eight critical risks in economic work in 2021.^136^

All these facts point to the conclusion that the Chinese government was keen to adopt rules to deal with new issues in the digital market and rapidly cope with problems to maintain social stability. But how does government achieve these goals?

#### The power of competition institutions

China is a big, complex country with a highly centralized system of government.^137^The powers of competition institutions can also be understood from three aspects: legislators, competition authorities and judicature.

First of all, the powers and responsibilities of state institutions are prescribed by Chapter 3 of the *Constitution*. Article 58 states that the National People's Congress (NPC) and its Standing Committee exercise the legislative power of the State.^138^ Article 85 prescribes that the State Council is the Central People’s Government and the executive organ of the highest state organ of power. It is the highest state administrative organ. Furthermore, Section 8 prescribes that the People’s Courts shall be responsible for the adjudicatory work. Based on China’s Constitution, the three powers of legislation, administration, and judicatory seem balanced and independent.

However, Article 9 of the *Chinese Legislation Law* also prescribes that the NPC and its Standing Committee can decide to authorize the State Council to formulate hard law named ‘administrative regulations’.^139^ According to this Article, the State Council has the administrative power to supervise digital markets, and has the legislative power delegated by the NPC and its Standing Committee. Similarly, a department of the State Council—the State Administration for Market Regulation (SAMR),^140^ can issue hard law named ‘rules’. Lastly, the State Council and the SAMR can also issue soft law instruments and apply these competition rules to individual cases.

#### Analysis

Based on the above, on the one hand, it is obvious that central competition authorities, the State Council and the SAMR, can issue both hard and soft law to regulate competition in the digital market. However, because the Chinese government has needed to rapidly cope with new problems to maintain social stability, soft law has been considered to be a better choice, in order to quickly respond to issues triggering massive public attention. As the Anti-Monopoly Commission of the State Council stressed in the Explanation of the Guidelines:

“The aims of the Guideline are to promote innovation, highlight problem-orientation and respond to public concerns”.^141^

On the other hand, Chinese competition authorities have both legislative and administrative powers. Due to the strong power of competition authorities, businesses cannot ignore soft instruments and believe they do not affect them. Therefore, China officially published the *Platform Guidelines* in February 2021. Since then, the SAMR made punishment decisions for ninety-two cases in the Internet sectors between March and December 2021.^142^ Besides, the SAMR and other three departments have published a hard law instrument named the *Provisions on the Administration of Algorithm Recommendations for Internet Information Services*,^143^ which has some provisions related to fair competition. However, it is out of scope of this paper, because it aims at regulating specific technology and not (or only indirectly) at assessing behaviors of tech firms under competition law.

Additionally, China has established the State Anti-Monopoly Bureau (SAMB) on 18 November 2021 to strengthen the power of competition authority and promote law enforcement. Wang Yong, head of the Anti-Monopoly Commission of the State Council, called on the Bureau to further implement the fair competition policies, and strengthen supervision and law enforcement in areas involving the platform economy and innovation of science and technology.^144^ In fact, it was SAMR’s anti-monopoly department that has been elevated to the deputy ministerial-level SAMB. The higher-ranking authority could assist antitrust investigators in receiving resources when investigating cases. Additionally, we assume that the establishment of SAMB could increase its influence on private sectors, and then promote the effectiveness of soft law enforcement. However, the effects of strengthening the competition authority’s power still need to be assessed.

## Comparing the choices of the EU and China

After reviewing the EU and China’s choice of regulatory tools, this part compares the EU and China’s choices from two aspects.

### Different conflicts and aims for regulating

The EU and China face problems that are not entirely the same in the digital market. Both the EU and China realized the importance of innovation and dynamic competition in developing the digital economy, which influenced their respective competition laws and policies. However, the Chinese digital market is dominated by native tech companies, while the European market is dominated by non-native companies from the US. That significant difference must influence their attitudes and aims towards Big Tech firms and regulations.

The EU aims to harmonize its policy aimed at digital markets and to promote European businesses. EU regards competition issues in the digital market as having an intrinsically cross-border nature. The Commission argued that regulatory initiatives by Member States could not fully address those challenges and effects; without action at the EU level, they could lead to a fragmentation of the Internal Market.^145^ More importantly, the conflict between promoting the European digital businesses and domination by international tech firms might lead to tough measures taken by the EU. For example, US critics particularly charge European regulators with attempting to institute a regime of economic protectionism that would give European businesses an unfair advantage.^146^ Some researchers also criticize that the EU overlooked the economic evidence that favors large platforms, and the *DMA* reveals a positive bias toward European SMEs.^147^ Based on the *DMA*, it is obvious that the Commission pays attention to the large platforms called ‘gatekeepers’. Without a doubt, these well-identified firms are just a handful of corporations, mostly American and Chinese.^148^

However, China is a state with a unified market. The Chinese government recognizes social stability as a top priority. When serious cases triggered massive public attention to the digital market, the State Council and SAMR rapidly took measures to deal with cases and quickly issued rules for guiding the native platform operators. Further, one of the major objectives of China is to set global standards and data-related technologies, the same as the US, due to their technological dominance.^149^ Although the Chinese government realized the importance of competition regulation to social stability, the main concern is that regulatory burdens are often perceived as a major obstacle to innovation. Thus, the main issue faced by the Chinese government would be how to balance the competition and innovation between native tech companies.

### Different power design of competition institutions

The competition institutions of the EU and China are essentially different. China is a state, while the EU is a supranational organization.

With a highly centralized system of government, hierarchical control plays a decisive role in China.^150^ The powers of the State Council and SAMR are conferred by the *Constitution*. Consistent enforcement of soft law by local competition authorities would not be a concern for China.^151^ In contrast, the EU’s powers were conferred by the Treaties agreed by all Member States. When Article 288 *TFEU* clarified that recommendations and opinions have no binding force, both the Courts and NCAs have no obligation to apply or directly apply soft law. Thus, the principles of legal certainty and consistency may be undermined by soft law’s uncertain workings in national legal orders—the legal vis-à-vis practical effects that those instruments generate are far from clear.^152^

Furthermore, Chinese competition authorities have both legislative and administrative powers to issue hard and soft laws, and then enforce their rules. In contrast, the Commission does not have legislative power, meaning it can only issue soft law, besides its important task of enforcing all rules. Obviously, Chinese competition authorities may enjoy stronger power and more room than the EU, leading Chinese competition authorities to have more approaches to influence tech firms when applying their rules. For instance, the Chinese central authority maintains extensive administrative discretionary power and is able to strategically leverage state media to influence firms under investigation.^153^ It is hard to imagine that private firms ignore soft law issued by Chinese competition authorities. Although some scholars criticize that the Commission is becoming more and more powerful nowadays, the Commission still cannot issue hard law by itself.

## Conclusions

Are there reasons for competition regulators to choose different legal instruments between hard and soft law? On the basis of the fact that the EU published two hard law instruments (the *P2B Regulation* and the *DMA*) while China published a soft law instrument (the *Platform Guidelines*) for the similar issue of fair competition in digital markets, this paper provides two main reasons that may be considered by regulators for using the different tools.

Firstly, the characteristics of digital markets show that the EU and China indeed face the not exactly the same challenges, which may influence their strategies in competition law and policies. The EU faces the cross-border nature of digital markets dominated by international tech firms and the regulatory fragmentation in the internal market, causing legal uncertainty. However, the Chinese digital market is dominated by native tech firms, which brought problems and triggered public concerns.

Secondly, the different power design of competition institutions shows that the Chinese competition authorities may have stronger powers and more rooms to influence tech firms compared to the Commission, because the Chinese competition authorities can issue both hard and soft law approaches while the Commission can only publish soft law.

Nevertheless, the findings of this article go beyond the reasons for the EU and China’s choices based on the pros and cons analysis of soft and hard law instruments. In light of the comparative analysis, this paper also explores the appropriateness of their different choices. We conclude that both the EU and China have made use of the advantages of soft or hard law while finding their ways to overcome the disadvantages.

On the one hand, if the EU want to protect its Internal Market from abusive practices by Big Tech firms, it seems the most appropriate to introduce hard law instruments at the EU level which directly apply in all Member States. Meanwhile, it is wise to add review articles to overcome inflexibility. However, the effects of the regular review still need to be assessed in practice to see whether it is flexible enough to cope with the issues in the digital market.

On the other hand, China has been able to take advantage of soft law to rapidly respond to public concerns and maintain social stability. Moreover, it has used the power of competition authorities to publish soft law and use it to guide and influence the behaviors of private firms. However, it is necessary to examine whether it is lawful and effective in promoting law enforcement by strengthening administrative power.

This paper does not suggest that hard law is better than soft law or the opposite. Indeed, a hybrid system with both hard and soft law is common in most legal regimes. However, the experience of China and the EU do suggest that the differences between the two regulatory tools should be known and considered during the legislative and policy-making processes.

## Footnotes

1 Zhang L, and Chen S, (Sep. 2019). IMF Working Paper: China’s Digital Economy Opportunities and Risks, 3. Available from: https://www.imf.org/en/Publications/WP/Issues/2019/01/17/Chinas-Digital-Economy-Opportunities-and-Risks-46459.

2 WTO, (2020). WTO Trade Report: Government Policies to Promote Innovation in the Digital Age. 20. Available from: https://onlinebookshop.wto.org/shop/article_details.asp?Id_Article=908.

3 United Nations Conference on Trade and Development, (2021). Digital Economy Report 2021: Cross-border data flows and development: For whom the data flow*.* 25. Available from: https://unctad.org/system/files/official-document/der2021_en.pdf.

4 Crémer J, Montjoye Y, and Schweitzer H, (2019). Competition Policy for the Digital Era (Report for the European Commission) 12. Available from: https://ec.europa.eu/competition/publications/reports/kd0419345enn.pdf.

5 Cohen J, (2019). *Between Truth and Power—The Legal Constructions of Informational Capitalism*. Oxford Scholarship Online. 173.

6 Crémer, Montjoye, and Schweitzer, *supra* note 4, at 13. See also, WTO, *supra* note 2, at 43.

7 European Competition Network, (2021), Joint paper of the heads of the national competition authorities of the European Union. 1. Available from: https://www.bundeskartellamt.de/SharedDocs/Publikation/EN/Others/DMA_ECN_Paper.pdf;jsessionid=74ACBB770482E787E4EDA9CE4A3C495B.1_cid378?__blob=publicationFile&v=2.

8 Cohen, *supra* note 5, at 175.

9 Ibid.

10 Kira B, (2019). ‘Competition Policy in the Global Connected Economy: A Promising Research Agenda’. *De Lege Ferenda*. 2.

11 Graef I, (2020). ‘Effective enforcement matters more than new rules in the EU’s efforts to regulate gatekeeping platforms’. Available from: https://promarket.org/2020/10/11/effective-enforcement-rules-eu-regulate-gatekeeping-platforms/. See also Cohen, *supra* note 5, at 179.

12 Ibid.

13 Cohen, *supra* note 5, at 174.

14 European Competition Network, (Jun. 2021). How national competition agencies can strengthen the DMA, Joint paper of the heads of the national competition authorities of the European Union. 3. Available from: https://www.bundeskartellamt.de/SharedDocs/Publikation/EN/Others/DMA_ECN_Paper.pdf?__blob=publicationFile&v=2.

15 Snyder F, (in press for 2023), 'Bamboo, or Governance through Soft Law: Hybridity, Legitimacy and Sustainability'*Edward Elgar Research Handbook on Soft Law* (M Eliantonio, E Korkea-aho and U Mörth (eds), Edward Elgar Publishers, Cheltenham UK). See also: Trubek D, Cottrell P, and Mark Nance M, (2005). *“Soft Law,” “Hard Law,” and European Integration: Toward a Theory of Hybridity.* Jean Monnet Working Paper 02/05. 31. Available from: https://ssrn.com/abstract=855447.

16 United Nations Conference on Trade and Development, *supra* note 3, at 4.

17 Cohen, *supra* note 5, at 187.

18 For example, Germany has amended the German Competition Act (GWB) to regulate the digital market.

19 Eifert M, Metzger A, Schweitzer H, and Wagner G, (2021). ‘Taming the giants: The DMA/DSA package’. Vol. 58 (4) *Common Market Law Review.* 989. Available from: https://kluwerlawonline.com/JournalArticle/Common+Market+Law+Review/58.4/COLA2021065.

20 King & Wood Mallesons, (2020). 10 Highlights of the Antitrust Guidelines for Platform Economy. Available from: https://www.chinalawinsight.com/2020/11/articles/compliance/10-highlights-of-the-antitrust-guidelines-forplatform-economy/ [https://perma.cc/ZJ2Z-YZQD].

21 Trubek D, and Trubek L, (2007), ‘New Governance and Legal Regulation: Complementarity, Rivalry, and Transformation’, *Columbia Journal of European Law,* 539. Available from: https://ssrn.com/abstract=988065. See also Wincott D, (2001). ‘Looking Forward or Harking Back? The Commission and the Reform of Governance in the European Union’, *Journal of Common Market Studies,* 905.

22 Maher I, (2007). ‘Regulation and Modes of Governance in EC Competition Law: What's New in Enforcement?’ *Fordham International Law Journal,* 1713. Available from: https://ir.lawnet.fordham.edu/ilj/vol31/iss6/6.

23 Stefan O, (2012). ‘Hybridity Before the Court: A Hard Look at Soft Law in the EU Competition and State Aid Case Law’. Vol. 37 *European Law Review,* 51.

24 Baxter R, (1980). ‘International Law in Her Infinite Variety’, Vol. 29 *The International and Comparative Law Quarterly,* 566.

25 Chinkin C, (1989). ‘The Challenge of Soft Law: Development and Change in International Law’. *The International and Comparative Law Quarterly.* 852.

26 Terpan F, (2015). ‘Soft Law in the European Union—The Changing Nature of EU Law’. Vol. 21 *European Law Journal,* 70.

27 Snyder F, (1993). ‘The Effectiveness of European Community Law: Institutions, Processes, Tools and Techniques’. Vol. 56 *The Modern Law Review.* 31.

28 Snyder, *supra* note 15. See also: Trubek, Cottrell, and Nance, *supra* note 15.

29 Abbott K and Snidal D, (2000). 'Hard and Soft Law in International Governance'. *International Organization* Vol. 54, *Legalization and World Politic*s. 434.

30 Cosma H and Whish R, (2003). ‘Soft Law in the Field of EU Competition Policy’. Vol. 14(1) *European Business Law Review,* 33.

31 Snyder, *supra* note 15. See also: Trubek, Cottrell, and Nance, *supra* note 15, at 15.

32 Ibid, at 15.

33 Ibid, at 31.

34 Ibid, at 31.

35 Abbott, and Snidal, *supra* note 29, at 423.

36 Ibid, at 453.

37 Jiang M, (2006). ‘软法的兴起与软法之治 (The rise of soft law and the governance of soft law)’, *中国法学China Legal Science.* 33.

38 Weeks G, (2016). *Soft law and public authorities: remedies and reform.* Hart Publishing. 44. see also, Jiang, *supra* note 37, at 33.

39 Cosma, and Whish, *supra* note 30, at 33.

40 Stefan O, Avbelj M, Eliantonio M, Hartlapp M, Korkea‐aho E, and Rubio N, (2019). ‘EU Soft Law in the EU Legal Order: A Literature Review’. 21. Available from: https://ssrn.com/abstract=3346629.

41 Snyder, *supra* note 15. See also: Trubek, Cottrell, and Nance, *supra* note 15, at 16.

42 Several researches focus on issuing and applying soft law during the COVID period. See as: Eliantonio M, Kerkea-Aho E and Vaughan S, (2021), ‘EJRR Special Issue Editorial: COVID-19 and Soft Law: Is Soft Law Pandemic-Proof?’ *European Journal of Risk Regulation* 1–6. Cheng X, (2021). ‘Soft Law in the Prevention and Control of the COVID-19 Pandemic in China: Between Legality Concerns and Limited Participatory Possibilities’. *European Journal of Risk Regulation.* 7–25.

43 Luo H, and Song G, (2009), *Soft Law Governance: Towards an Integrated Approach* (Law Press China Publishing, 293.

44 Jiang, *supra* note 37, at 3.

45 Snyder, *supra* note 15. See also: Trubek, Cottrell and Nance, *supra* note 15, at 16.

46 Jiang M, (2014).‘改革法治与国家治理现代化 (Reform of the Rule of Law and Modernization of National Hovernance)’. 中共中央党校学报 Journal of the Party School of the Central Committee of the C.P.C, 53.

47 Luo H and Song G, (2006). ‘认真对待软: 公域软法的一般理论及其中国实践 (Take soft law seriously—The General Theory of Public Domain Soft Law and Its Practice in China)’. *中国法学China Legal Science.* 7.

48 Snyder, *supra* note 15. See also: Trubek, Cottrell and Nance, *supra* note 15, at 6.

49 Weeks, *supra* note 38, at 44.

50 Cosma and Whish, *supra* note 30, at 34.

51 Ibid.

52 European Parliament, Resolution of the European Parliament ‘on the institutional and legal implications of the use of soft law instruments’. 2007/2028(INI). 1.

53 Raz J, (1972), ‘Legal Principles and the Limits of Law’. *Yale Law Journal.* 841.

54 Braithwaite J, (2002). ‘Rules and Principles: A Theory of Legal Certainty’. *Australian Journal of Legal Philosophy.* 50. Available from: https://ssrn.com/abstract=329400.

55 Ibid, at 54.

56 Ibid, at 54.

57 Stefan, Avbelj, Eliantonio, Hartlapp, Korkea‐aho and Rubio, *supra* note 40, at 14.

58 Reinicke W, and Witte J, (2000). ‘Interdependence, Globalization, and Sovereignty: The Role of Non-Binding Legal Accords’. *Commitment and Compliance: The Role of Non-Binding Norms in the International Legal System* (Oxford University Press). 94–95.

59 Howells G, (1999). ‘“Soft law” in EC consumer law’. *Lawmaking in the European Union*. Craig P and Harlow C (eds). Chapter 15.

60 Stefan, Avbelj, Eliantonio, Hartlapp, Korkea‐aho, and Rubio, *supra* note 40, at 14.

61 Cosma, and Whish, *supra* note 30, at 34.

62 Braithwaite, *supra* note 54, at 47.

63 Snyder, *supra* note 15. See also: Trubek, Cottrell and Nance, *supra* note 15, at 6.

64 Luo H, (2013). ‘中国法治要软硬并举 (The rule of law in China needs both hard law and soft law)’. *工商行政管理Administration for Industry and Commerce.* 5. See also: Luo, and Song, *supra* note 45, at 214.

65 Hodson D, and Maher I, (2002), ‘European Monetary Union: Balancing Credibility and Legitimacy in an Asymmetric Policy Mix’, *Journal of European Public Policy*. 6. See also, Trubek, Cottrell, and Nance, *supra* note 15, at 32.

66 Luo, and Song, *supra* note 47, at 5.

67 Snyder, *supra* note 15. See also: Trubek, Cottrell, and Nance, *supra* note 15, at 21.

68 Ibid.

69 United Nations Conference on Trade and Development, (Geneva 2019). Digital Economy Report 2019: Value Creation and Capture: Implications for Developing Countries. 37. Available from: https://unctad.org/webflyer/digital-economy-report-2019#tab-2.

70 Ezrachi A, (2021). *Competition and Antitrust Law: A Very Short Introduction*. Oxford University Press. 33.

71 Dunne N, (2015). *Competition law and economic regulation making and managing markets*. Cambridge University Press. 21.

72 Frenz W, (2016). *Handbook of EU Competition Law.* Springer Berlin Heidelberg. 4.

73 European Commission, (2020c). Shaping the digital transformation in Europe. 7. Available from: https://data.europa.eu/doi/10.2759/294260.

74 Ibid, at 3.

75 European Commission, Proposal for the Digital Market Act (2020). 1. Available from: https://ec.europa.eu/info/sites/default/files/proposal-regulation-single-market-digital-services-digital-services-act_en.pdf.

76 Ibid. The US and China, notably the world’s two largest national economies, appear to have a head start in digital uptake and investment.

77 Ibid, at 18.

78 Germany Monopolies Commission, (Jun. 2015). Competition policy: The challenges of digital markets. Special Report by the Monopolies Commission pursuant to Section 44(1)(4) of the Act Against Restraints on Competition, 53. Available from: https://www.monopolkommission.de/index.php/en/press-releases/52-competition-policy-the-challenge-of-digital-markets.

79 Ibid.

80 Website of Statcounter. The last access on 29 March 2022. Available from: https://gs.statcounter.com/search-engine-host-market-share/all/europe/#monthly-202101-202112-bar.

81 European Commission, *supra* note 75, at 2.

82 Ibid.

83 The Regulatory Scrutiny Board is an independent body within the Commission to provide central quality control and support for Commission impact assessments and evaluations at early stages of the legislative process. Its Opinion was published by the Commission together with other official documents in the website. Available from: https://ec.europa.eu/info/law/law-making-process/regulatory-scrutiny-board_en.

84 European Commission, (2020b). Regulatory Scrutiny Board Opinion for the Digital Market Act (Final). Available from: https://ec.europa.eu/competition-policy/system/files/2021-09/DMA_opinion_of_the_board.pdf. See also: Portuese A, (2021). ‘The Digital Markets Act: European Precautionary Antitrust’. *Information Technology,* 9–10, 21–44. Petit N, (2021). ‘The Proposed Digital Markets Act (DMA): A Legal and Policy Review’. *Journal of European Competition Law & Practice.* 529–541.

85 European Commission, Commission Staff Working Document, Impact Assessment Report of DMA, (Dec. 2020), 14. Available from: https://ec.europa.eu/competition-policy/system/files/2021-09/DMA_impact_assessment_part1.pdf.

86 Ibid.

87 European Commission, *supra* note 75, at 5.

88 On 18 January 2021, the German legislature adopted the *Tenth Amendment to the German Competition Act (‘Gesetz gegen Wettbewerbsbeschränkungen’*). Available from: https://www.gesetze-im-internet.de/englisch_gwb/englisch_gwb.pdf.

89 Franck J, and Peitz M, (2019). ‘Digital Platforms and the New 19a Tool in the German Competition Act1’. Vol. 12 *Journal of European Competition Law & Practice.* 513–528.

90 European Commission, *supra* note 75, at 8. See also, European Commission, (Dec.2020a), Commission Staff Working Document Executive Summary of the Impact Assessment Report. 1. Available from: https://ec.europa.eu/competition-policy/system/files/2021-09/DMA_summary_of_impact_assessment.pdf.

91 European Commission, *supra* note 75.

92 European Commission, (Sep. 2020c). Shaping the digital transformation in Europe, 18–19. Available from: https://data.europa.eu/doi/10.2759/294260.

93 European Commission, *supra* note 85, at 111.

94 Schrepel T, (2021). *Blockchain* + *Antitrust*, Edward Elgar Publishing, Chapter 2: European Union: European Commission. 7.

95 Snyder F, (2021). ‘Three Worlds of Chinese Soft Law’, *EU Soft Law in the Member States: Theoretical Findings and Empirical Evidence* (Ed. M Eliantonio, E Korkea-aho and O Stefan, Oxford: Hart Publishing), 279.

96 Chalmers D, Davies G, and Monti G, (2019). *European Union Law.* Fourth Edition Cambridge Publish. 70.

97 Craig P, and Búrca G, (2011). *EU Law**: **Text, Cases, and Materials, 5th Edition* Oxford University Press. 128.

98 Wiggers M, Struijlaart R, and Dibbits J, (2019). *Digital Competition Law in Europe – A Concise Guide.* Kluwer Law International. 4.

99 Including Regulation 17, the initial legislation setting out the basic rules of procedure implementing Articles 101 and 102 TFEU. Available from: https://eur-lex.europa.eu/legal-content/EN/ALL/?uri=celex%3A31962R0017. Regulation 19/65 on the application of Article 101(3) of the Treaty to certain categories of agreements and concerted practices, and amended by Regulation 1215/1999. Available from: https://eur-lex.europa.eu/legal-content/EN/LSU/?uri=celex:31965R0019. The Merger Regulation and the Regulation on Procedure, which replaced Regulation 17 with effect as of 1 May 2004. Available from: https://ec.europa.eu/competition-policy/mergers/procedures_en.

100 Wiggers, Struijlaart, and Dibbits, *supra* note 98, at 1.

101 Ibid.

102 Ibid, at 9.

103 Senden L, (2004). *Soft Law in European Community Law*. Hart Publishing. 118–119.

104 For example, Senden, *supra* note 103, at 361; See also Stefan O, (2008), ‘European Competition Soft Law in European Courts: A Matter of Hard Principles’, Vol. 14. *European Law Journal,* 753–772.

105 Chalmers, Davies, and Monti, *supra* note 96, at 368–369.

106 Eliantonio M, (2020). ‘Judicial Review of Soft Law before the European and the National Courts—A Wind of Change Blowing from the Member States?’ *EU Soft Law in the Member States: Theoretical Findings and Empirical Evidence* (Edited by M Eliantonio, E Korkea-aho and O Ştefan). 284. See also: Judgment of the Court (Grand Chamber) of 15 July 2021, Fédération bancaire française (FBF) v Autorité de contrôle prudentiel et de résolution (ACPR), Case C-911/19, https://eur-lex.europa.eu/legal-content/GA/TXT/?uri=CELEX:62019CJ0911.

107 https://eur-lex.europa.eu/legal-content/EN/ALL/?uri=celex%3A31962R0017.

108 Wiggers, Struijlaart and Dibbits, *supra* note 98, at 6.

109 Cseres K, and Outhuijse A, (2017). ‘Parallel enforcement and accountability: the case of EU competition law’, *Law Enforcement by EU Authorities: Implications for Political and Judicial Accountability.* 82.

110 Petit N, and Rato M, (2009), From Hard to Soft Enforcement of EC Competition Law – A Bestiary of ‘Sunshine’ Enforcement Instruments in Alternative Enforcement Techniques in EC Competition Law: Settlements, Commitments and Other Novel Instruments. 203.

111 Georgieva Z, (2017). *Soft Law in EU Competition Law and its Reception in Member States’ Courts—An Empirical Study on National Judicial Attitudes to Atypical Legal Instruments in EU Competition Law.* 25.

112 Wiggers, Struijlaart, and Dibbits, *supra* note 98, at 18.

113 Georgieva, *supra* note 111, at 22.

114 Whish R, and Bailey D, (2021), *Competition Law*, 10th ed. Section 2 Oxford University Press, 57.

115 Ibid.

116 For example, the research of Eliantonio and Alberti illustrates that Italian national courts make frequent use of EU soft law and endow it with binding effects in some policy fields, such as competition and State aid. See from Alberti J, and Eliantonio M, (2021). ‘Judges, public authorities and EU soft law in Italy—How you cannot tell a book by its cover’. *EU soft law in the member states: Theoretical findings and empirical evidence*. Oxford: Hart Publishing. 198.

117 For example, Giulia Gentile’s research shows that French courts have not taken a clear stance on the effects of national soft law measures implementing EU soft law and have not interpreted the national acts in light of the original EU measures. See Gentile G, (2021). ‘Verba Volant, Quoque (Soft Law) Scripta?’: An Analysis of the Legal Effects of National Soft Law Implementing EU Soft Law in France and the UK’, *EU Soft Law in the Member States: Theoretical Findings and Empirical Evidence*, Ed. M Eliantonio, E Korkea- aho and O Stefan. Oxford: Hart Publishing. 95.

118 Georgieva, *supra* note 111, at 22.

119 Ibid.

120 Chalmers, Davies, and Monti, *supra* note 96, at 61.

121 European Commission, *supra* note 75, at 5.

122 Regulation (EU) 2022/1925 of the European Parliament and of the Council on Contestable and Fair Markets in the Digital Sector and Amending Directives (EU) 2019/1937 and (EU) 2020/1828 (Digital Markets Act). *Official Journal of the European Union*, L265/1. Available from: https://eur-lex.europa.eu/legal-content/EN/TXT/?uri=CELEX%3A32022R1925.

123 Shang M, (2009). ‘Antitrust in China – A Constantly Evolving Subject’. *Competition Law International* 4–5.

124 Ibid.

125 Zhang H, (2021), *Chinese Antitrust Exceptionalism*, Oxford University Press, 40.

126 Zhang H, (2022), ‘Agility Over Stability: China’s Great Reversal in Regulating the Platform Economy’, *Harvard International Law Journal,* 4. Available from: https://papers.ssrn.com/sol3/papers.cfm?abstract_id=3892642.

127 Zhang, and Chen, *supra* note 1, at 4.

128 Ibid, at 7.

129 Chinese Cyberspace Administration, *14th Five-Year National Information Plan* (in Chinese). Available from: http://www.cac.gov.cn/2021-12/27/c_1642205314518676.htm. The last access on 5 March 2022.

130 United Nations Conference on Trade and Development, *supra* note 2, at 23.

131 Website of Statcounter. The last access on 29 March 2022. Available from: https://gs.statcounter.com/search-engine-market-share/all/china/#monthly-202101-202112-bar

132 McMorrow R, and Liu N, (Jan. 2020), Sellers asked to choose in battle between Alibaba and Pinduoduo, *Financial Times.* Available from: https://www.ft.com/content/b55d0e0a-33a1-11ea-9703-eea0cae3f0de

133 Instant price increase of 250 yuan to buy air tickets? (in Chinese), *China Consumer News*. (Oct. 2021). Available from: https://baijiahao.baidu.com/s?id=1714261580680873026&wfr=spider&for=pc

134 Zhang, *supra* note 126, at 18.

135 Dong J, (14 December 2020). China: Understanding policy signals of the December CPC Politburo meeting. *BBVA Research*. Available from: https://www.bbvaresearch.com/en/publicaciones/china-understanding-policy-signals-of-the-december-cpc-politburo-meeting/ See also: The Think Tank, (14 December 2020). 中央政治局会议最新解读 (The latest interpretation of the Politburo meeting) (in Chinese). *The Paper*. Available from: https://m.thepaper.cn/baijiahao_10394098.

136 Xinhua News Agency, (19 December 2020). 中央经济工作会议的七大民生看点(Seven highlights of the Central Economic Work Conference) (in Chinese). Website of China State Council. Available from: https://www.gov.cn/xinwen/2020-12/19/content_5571111.htm.

137 Wang J, (2021). ‘Judicial Review of Regulatory Documents in Administrative litigation in Administrative Litigation in China’. *University of Pennsylvania Asian Law Review.* 337. Available from: https://scholarship.law.upenn.edu/alr/vol16/iss2/5.

138 http://www.npc.gov.cn/englishnpc/constitution2019/201911/1f65146fb6104dd3a2793875d19b5b29.shtml. The last access on 24 April 2022.

139 http://www.npc.gov.cn/zgrdw/englishnpc/Law/2007-12/11/content_1383554.htm. The last access on 24 April 2022. Except for the affairs concerning criminal offences and their punishment, mandatory measures and penalties involving deprivation of citizens of their political rights or restriction of the freedom of their person, and the judicial system.

140 And the new department: State Anti-Monopoly Bureau (SAMB).

141 Anti-Monopoly Commission of the State Council, (2021).《国务院反垄断委员会关于平台经济领域的反垄断指南》解读 (Explanation of the Anti-monopoly Guidelines of the Anti-Monopoly Committee of the State Council on Platform Economy) (in Chinese). Available from: https://gkml.samr.gov.cn/nsjg/xwxcs/202102/t20210207_325970.html.

142 https://www.sohu.com/a/509453070_161795 (in Chinese), The last access on 24 April 2022. See also: https://www.163.com/dy/article/GRF3DD5F05129QAF.html (in Chinese), The last access on 24 April 2022.

143 The website of Cyberspace Administration of China (in Chinese). The last access on 23 June 2022. http://www.cac.gov.cn/2022-01/04/c_1642894606364259.htm.

144 The website of the State Council (In English), The last access on 20 January 2022. http://english.www.gov.cn/statecouncil/wangyong/202111/19/content_WS61973e4cc6d0df57f98e52d9.html.

145 European Commission, *supra* note 75, at 3.

146 Cohen, *supra* note 5, at 178.

147 Portuese, *supra* note 84, at 84.

148 Ibid, at 21.

149 United Nations Conference on Trade and Development, *supra* note 3, at 113.

150 Wang, *supra* note 137, at 337.

151 For example, Beijing competition authority published “北京市平台经济领域反垄断合规指引 (Anti-monopoly compliance guidelines in the field of platform economy in Beijing)”in 2021, based on the Guideline issued by central competition authority. (in Chinese). Available from: http://scjgj.beijing.gov.cn/zwxx/scjgdt/202112/t20211209_2557026.html.

152 Georgieva, *supra* note 111, at 8.

153 Zhang, *supra* note 125, at 79.
